# A non-linear association between low-density lipoprotein cholesterol and the risk of diabetic kidney disease in patients with type 2 diabetes in China

**DOI:** 10.1016/j.pmedr.2024.102840

**Published:** 2024-07-27

**Authors:** Xi Xiang, Guangming Chen, Yongjun Ma, Huabin Wang

**Affiliations:** a Department of Clinical Laboratory, Affiliated Jinhua Hospital, Zhejiang University School of Medicine, Jinhua City, Zhejiang Province 321000, China; b Department of General Practice, Affiliated Jinhua Hospital, Zhejiang University School of Medicine, Jinhua, China

**Keywords:** Low-density lipoprotein cholesterol, Non-linear association, Diabetes kidney disease, Type 2 diabetes, Restricted cubic spline curves

## Abstract

**Objective:**

To explore the intrinsic relationship between low-density lipoprotein cholesterol (LDL-C) and diabetic kidney disease (DKD) in patients with type 2 diabetes (T2D) in China.

**Methods:**

This cross-sectional study included 1,313 patients with type 2 diabetes treated at the Affiliated Jinhua Hospital of Zhejiang University School of Medicine, located in Jinhua, China. The data were combined from two periods, 2017 and 2020–2021. Participants were categorized into groups with and without DKD. The relationship between LDL-C levels and DKD was evaluated employing logistics regression analysis and restricted cubic spline (RCS) curves.

**Results:**

Generally, there was no statistical difference in LDL-C levels between DKD and non-DKD groups, however, a significantly non-linear relationship (P_non-linear_ = 0.011) was observed between LDL-C levels and DKD prevalence after adjusting for confounding factors according to the RCS analysis. Two optimal cut-points of 2.97 and 3.61 mmol/L were selected out using random forest algorithm. With the middle LDL-C concentration (2.97–3.61 mmol/L) as the reference, the odds ratios for low (<2.97 mmol/L) and high (>3.61 mmol/L) concentrations were 1.45 (1.08–1.96) and 1.47 (1.01–2.15) respectively, after adjusting for confounding factors in the multivariate analyses. Notably, this association was more pronounced among female participants in the subgroup analyses.

**Conclusion:**

A non-linear association was observed between LDL-C levels and the risk of DKD in patients with T2D in China. LDL-C levels below 2.97 mmol/L may elevate the risk of DKD, particularly in female patients with T2D.

## 1 Introduction

Diabetic kidney disease (DKD) is a prevalent complication of diabetes mellitus, placing a significant burden on global healthcare systems (Sinha and Nicholas, 2023, Liang et al., 2024, Sun et al., 2023). It is characterized by albuminuria, declining glomerular filtration rate, and ultimately, end-stage renal disease. The prevalence of DKD is increasing, with an estimated 30–40 % of individuals with type 1 or type 2 diabetes developing this disease during their lifetime (Saran et al., 2017, Durcan et al., 2022, Chen et al., 2023). Presently, DKD has emerged as the leading cause of the end-stage renal disease worldwide (Chen et al., 2023). DKD is associated with an increased risk of cardiovascular events, progression to kidney failure, and mortality (Levey et al., 2011, Singh et al., 2023), underscoring the importance of early detection and management of this condition. The progression to DKD depends on multiple factors, including genetic predisposition, glycemic control, blood pressure management, and lifestyle factors. Clinical guidelines recommend multiple strategies to prevent and manage DKD. These strategies primarily involve enhancing lifestyle habits, maintaining strict glycemic control, regulating blood pressure, and utilizing angiotensin-converting enzyme inhibitors (ACEI) or angiotensin receptor blockers (ARB) to decrease proteinuria and slow the advancement of kidney disease (Chinese Diabetes Society, 2023, de Sá et al., 2022). However, despite endeavors to reach the recommended targets for blood glucose and blood pressure, the persistent high risk of DKD persists (Russo et al., 2016, Li et al., 2023), highlighting the ongoing challenge of managing and preventing the progression of DKD.

Besides hyperglycemia and hypertension, multiple animal and epidemiological studies have demonstrated that dyslipidemia plays an important role in the DKD development and progression (Zhou et al., 2023, Macisaac et al., 2014, Kelsey et al., 2022). Among the individuals with type 2 diabetes (T2D), dyslipidemia is typically characterized by elevated levels of low-density lipoprotein cholesterol (LDL-C) and triglycerides (TG), as well as reduced levels of high-density lipoprotein cholesterol (HDL-C) (Lu et al., 2022, Taskinen, 2003). Statins, the primary agents in contemporary lipid-lowering therapy, lower plasma levels of LDL-C and TG by inhibiting cholesterol synthesis (Lu et al., 2022, Bussolati et al., 2005). A multicenter retrospective cohort study (Zhou et al., 2023) revealed that initiating statin therapy was linked to a decreased risk of kidney disease progression, especially in individuals maintaining stringent LDL-C control. However, some population-based studies (Nielsen and Nordestgaard, 2014, Hanai et al., 2017) indicated that the use of statin did not decrease the risk of kidney diseases in patient with diabetes. Considering these divergent outcomes, we speculated that the risk of DKD may not decrease with reductions in LDL-C or TG levels, suggesting a complex non-linear relationship, particularly between the DKD risk and LDL-C levels. Hence, we conducted the present study to determine the intrinsic association between the levels of LDL-C and DKD in patients with T2D in China.

## 2 Materials and methods

### 2.1 Study population

**Fig. 1:**
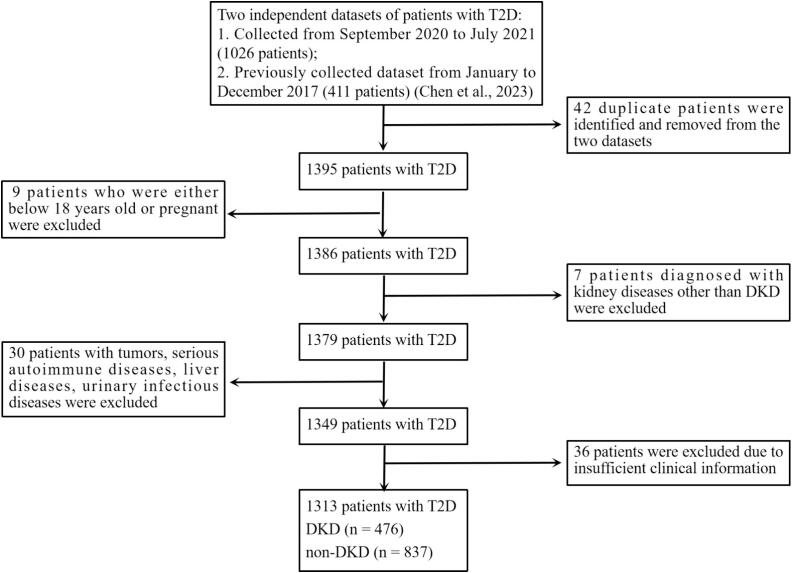
Flowchart of study participants' enrollment from a two-phase dataset of patients with T2D in Jinhua, China, from 2017 and 2020–2021. T2D: type 2 diabetes; DKD: diabetes kidney disease.

In this cross-sectional observational study, the study population comprised 936 patients with T2D who sought treatment at the Endocrinology Department of the Affiliated Jinhua Hospital, Zhejiang University School of Medicine between September 2020 and July 2021, and 411 participants diagnosed with T2D who had visited the same department from January to December 2017 (Chen et al, 2023). Following the removal of duplicate entries, these cohorts were merged to create the final study population, totaling 1313 individuals. The exclusion criteria of the study subjects (Chen et al, 2023) were as follows: (1) aged below 18 years old; (2) patients who were pregnant or diagnosed with type 1 diabetes; (3) patients diagnosed with kidney diseases other than DKD; (4) patients with tumors, serious autoimmune diseases, liver diseases, and/or urinary infectious diseases; (5) individuals with missing critical clinical parameters. See Fig. 1. The participants in this study had an average age of 59.20 ± 13.10 years, with a median diabetes duration of 8 years. Females comprised 41.20 % (541/1313) of the population, while subjects with hypertension accounted for 53.01 % (696/1313). The present study met the institution’s guidelines for protection of human subjects concerning their safety and privacy, and was approved by the Ethics Committee of Affiliated Jinhua Hospital, Zhejiang University School of Medicine.

### 2.2 Data collection

In the present study, the clinical characteristics of the participants were collected from the electronic health record system following their diagnosis of diabetes, during visits to the endocrinology department. The collected data included demographics and clinical parameters such as age, gender, height, weight, duration of diabetes (from the time of diagnosis), hypertension status, systolic blood pressure (SBP), diastolic blood pressure (DBP), angiotensin converting enzyme inhibitor (ACEI) or angiotensin receptor blocker (ARB) medication usage, and other variables. Laboratory parameters of the subjects were extracted from the examination system of Department of Clinical Laboratory. All blood samples from the patients were collected in the morning from fasting serum or whole blood, and urine samples were obtained from the first morning void. Moreover, all analyses were conducted on the same day the samples were collected. LDL-C, HDL-C, TG, total protein, serum albumin, uric acid, serum creatinine, urine albumin, and urine creatinine were analyzed using the Beckman Coulter automatic biochemical analyzer and its corresponding reagents. The measurement of glycated hemoglobin (HbA1c) levels were analyzed using high-performance liquid chromatography with the BIO-RAD D-100 analyzer.

The urine albumin-to-creatinine ratio (ACR) was calculated from the concentrations of urine albumin and urinary creatinine. The estimated glomerular filtration rate (eGFR) was determined by applying the Xiangya equation based on the serum creatinine levels (Xu et al., 2021, Li et al., 2019). The participants’ body mass index (BMI) was calculated based on their height and weight. In this study, DKD was defined as eGFR<60 ml/min/1.73 m^2^ and/or ACR>30 mg/g for at least three months caused by diabetes.

### 2.3 Statistical analysis

In this study, all data analyses and graph creation were conducted using R software version 4.3.2, SPSS statistical software version 26.0, and GraphPad Prism 8. The clinical characteristics of the subjects, categorized by DKD status, were presented as frequencies (percentages), mean ± SD, and median (25th and 75th percentiles) for categorical variables, normally and skewed continuous variables, respectively. Between-group comparisons were conducted by performing Pearson’s chi-squared tests, unpaired t-tests, and Mann-Whitney U tests. The levels of LDL-C levels were categorized into four groups by quartiles: Q1: 0.67–2.29 mmol/L, Q2: 2.30–2.88 mmol/L, Q3: 2.89–3.44 mmol/L, and Q4: 3.45–7.08 mmol/L. Violin plots were employed to observe the differences in DKD prevalence, ACR levels, and eGFR levels among LDL-C quartiles; in addition, prior to the comparative analyses, the levels of ACR underwent a natural logarithm transformation to enhance normality. Logistic regression analysis and restricted cubic spline (RCS) curve analysis were performed to evaluate the associations between the risk of DKD and the levels of LDL-C after adjusting for confounding factors. The random forest algorithm was used to determine the optimal cutoff points for LDL-C levels in relation to the risk of DKD. The continuous LDL-C data were divided into ten intervals (bins) to facilitate processing by the algorithm. The random forest model was trained using 2000 trees to ensure the stability and accuracy of the model. After the model training was completed, the most influential LDL-C intervals were identified based on the importance scores of features within the model. Subsequently, the midpoint values of these intervals were calculated to serve as potential cutoff points for changes in DKD risk. Furthermore, a threshold effect analysis of LDL-C levels on DKD risk using logistic regression was conducted. Finally, in this study, since 459 participants only had a single measurement of ACR and eGFR, we conducted sensitivity analysis to test whether the same non-linear relationship was observed in the 854 patients who were clearly classified according to DKD criteria. A value of *P*<0.05 was defined as statistical significance.

## 3 Results

### 3.1 Characteristics of the study population

Among the 1313 subjects with T2D, the prevalence of DKD was 36.25 % (476/1313). As shown in Table 1, compared to participants without DKD, patients with DKD were older, had higher SBP, higher BMI, a higher prevalence of hypertension, a higher rate of ACEI/ARB medication usage, a longer duration of diabetes, higher levels of HbA1c, TG, uric acid, Serum creatinine, serum albumin, and ACR, and lower eGFR levels. There were no statistically significant differences detected in gender distribution, DBP, HDL-C, and total protein levels between the two groups. Notably, no significant differences were observed in LDL-C levels between participants with and without DKD as well.

**Table 1:** Clinical characteristics of patients with T2D in Jinhua, China, combining data from 2017 and 2020–2021.

Variables	Overall (n = 1313)	Patients without DKD (n = 837)	Patients with DKD (n = 476)	P
Age (year)	59.20 ± 13.10	57.65 ± 12.21	61.94 ± 14.12	<0.001
Female, n (%)	541 (41.20)	339 (40.50)	202 (42.44)	0.493
SBP (mmHg)	137 ± 19	135 ± 18	142.023 ± 21.012	<0.001
DBP (mmHg)	79 ± 12	79 ± 11	79 ± 13	0.937
Hypertension, n (%)	696 (53.01)	362 (43.25)	334 (70.17)	<0.001
ACEI/ARB use, n (%)	491 (37.94)	273 (32.93)	218 (46.88)	<0.001
Diabetic duration (year)	8 (2, 13)	6 (2, 11)	10 (4, 16)	<0.001
BMI	24.72 ± 3.67	24.56 ± 3.63	25.00 ± 3.73	0.039
HbA1c (%)	8.47 ± 2.31	8.34 ± 2.24	8.70 ± 2.41	0.008
LDL (mmol/l)	2.91 ± 0.89	2.94 ± 0.83	2.85 ± 0.99	0.115
HDL (mmol/l)	1.17 ± 0.33	1.18 ± 0.33	1.16 ± 0.34	0.242
TG (mmol/l)	1.44 (1.03, 2.07)	1.41 (1.01, 2.03)	1.51 (1.06, 2.17)	0.019
Uric acid (μmol/L)	303 (254, 369)	291 (248, 349)	337 (267, 409)	<0.001
Serum creatinine (μmol/L)	73 (63, 85)	69 (61, 78)	82 (68, 103)	<0.001
Total protein (g/L)	65.74 ± 6.01	65.76 ± 5.40	65.71 ± 6.95	0.903
Serum albumin (g/L)	39.86 ± 4.53	40.49 ± 3.69	38.73 ± 5.53	<0.001
ACR (mg/g)	16.31 (7.01, 50.93)	8.94 (4.82, 15.34)	95.35 (46.53, 421.09)	<0.001
eGFR (ml/min/1.73 m2)	76.87 ± 12.47	80.31 ± 9.21	70.83 ± 14.91	<0.001

T2D: type 2 diabetes; DKD: diabetes kidney disease; SBP: systolic blood pressure; DBP: diastolic blood pressure; BMI: body mass index; ACEI: angiotensin converting enzyme inhibitor; ARB: angiotensin receptor blocker; HbA1c: glycated hemoglobin; LDL-C: low-density lipoprotein cholesterol; HDL-C: high-density lipoprotein cholesterol; TG: triglyceride; eGFR: estimated glomerular filtration rate; ACR: albumin-to-creatinine ratio.

### 3.2 The ACR, eGFR levels, and DKD prevalence among LDL-C quartiles

**Fig. 2:**
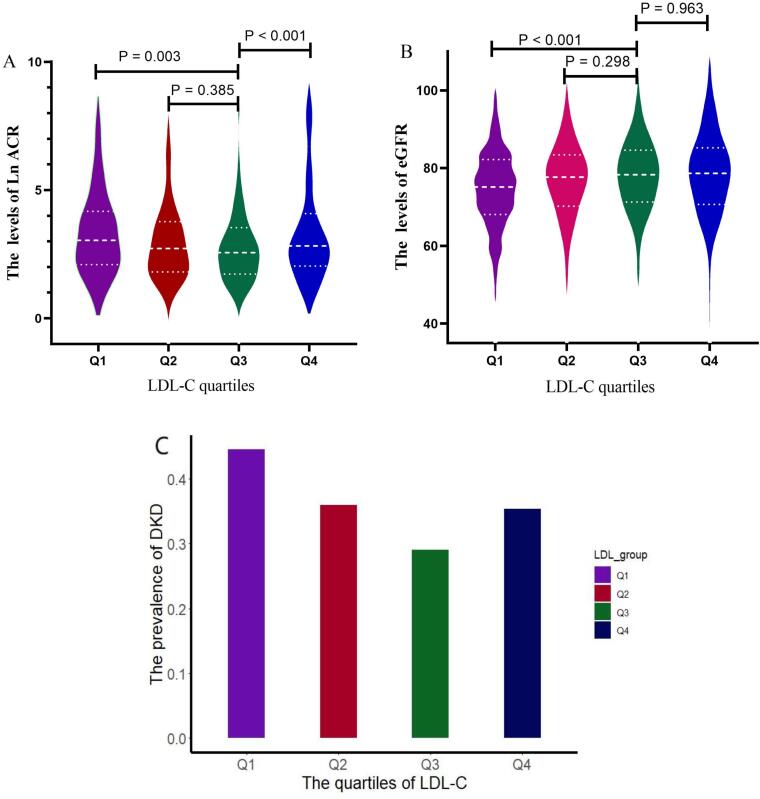
Distribution of ACR, eGFR, and DKD prevalence by LDL-C quartiles among patients with T2D in Jinhua, China, combining data from 2017 and 2020–2021. T2D: type 2 diabetes; DKD: diabetes kidney disease; LDL-C: low-density lipoprotein cholesterol eGFR: estimated glomerular filtration rate; ACR: albumin-to-creatinine ratio.

The levels of ACR, eGFR, and the prevalence of DKD across quartiles of LDL-C levels were evaluated (Fig. 2). In general, varying levels of ACR, eGFR, and the prevalence of DKD were observed among the quartiles of LDL-C levels (Q1, Q2, Q3, and Q4). Relatively, patients in the LDL-C Q3 quartile exhibited the lowest ACR levels and DKD prevalence, alongside the highest eGFR levels. Although the ACR levels in patients in the LDL-C Q3 quartile were slightly lower than those in Q2, the difference was not statistically significant; however, the ACR levels in patients in the LDL-C Q3 quartile were significantly lower than those Q1 and Q4 quartiles (*P*<0.05). The mean eGFR values for the patients in quartiles Q1, Q2, Q3, and Q4 were 74.34 ± 12.95, 77.06 ± 12.43, 78.07 ± 11.06, and 78.02 ± 12.93, respectively. Although the eGFR levels in patients in the Q3 quartile of LDL-C were slightly higher than those in the Q2 and Q4 quartiles, the differences were not statistically significant; however, the eGFR levels in the Q3 quartile were significantly higher than those in the Q1 quartile. The prevalence of DKD in patients with Q1, Q2, Q3, and Q4 quartiles of LDL-C was 44.54 %, 35.95 %, 29.01 %, and 35.37 %, respectively, with patients in the Q1 and Q3 quartiles having the highest and lowest DKD prevalence, respectively.

### 3.3 Association of LDL-C level with DKD based on logistic regression

Table 2 listed the relationship of LDL-C levels with the risk of DKD in patients with T2D. When LDL-C levels were analyzed as a continuous variable, no statistical association was found between LDL-C levels and the risk of DKD in either univariate or multivariate logistic regression analyses. However, when LDL-C levels were categorized into four quartiles, the DKD risk linked to LDL-C levels in Q2, Q3, and Q4 was significantly lower compared to that associated with Q1 (*P*<0.05). Notably, the lowest DKD risk was observed in Q3. After adjustment for age, gender, BMI, hypertension, diabetic duration, ACEI/ARB usage, HbA1c, HDL-C, and TG, the DKD risk related to LDL-C levels in Q3 remained the lowest [0.560 (0.409–0.766), P=0.006].

**Table 2:** Association between LDL-C levels and DKD risk among patients with T2D in Jinhua, China, combining data from 2017 and 2020–2021.

Variables	Univariate analyses		Multivariate analyses#	
	OR (95 % CI)	P value	OR (95 % CI)	P value
LDL-C (mmol/L)				
Continuous	0.90 (0.79–1.02)	0.099	0.93 (0.81–1.08)	0.355
Q1 (0.67–2.29)	1.0 (reference)	−	1.0 (reference)	−
Q2 (2.30–2.88)	0.70 (0.51–0.96)	0.024	0.81 (0.57–1.14)	0.216
Q3 (2.89–3.44)	0.51 (0.37–0.70)	< 0.001	0.60 (0.42–0.86)	0.006
Q4 (3.45–7.08)	0.68 (0.50–0.93)	0.016	0.82 (0.57–1.18)	0.278
P for trend	< 0.001		0.053	

# This analysis was adjusted for age, gender, body mass index, hypertension, diabetic duration, angiotensin converting enzyme inhibitor/angiotensin receptor blocker usage, glycated hemoglobin, high-density lipoprotein cholesterol, and triglycerides. T2D: type 2 diabetes; DKD: diabetes kidney disease; LDL-C: low-density lipoprotein cholesterol; OR: odds ratio.

### 3.4 Non-linear relationship of LDL-C levels with DKD based on RCS analysis

**Fig. 3:**
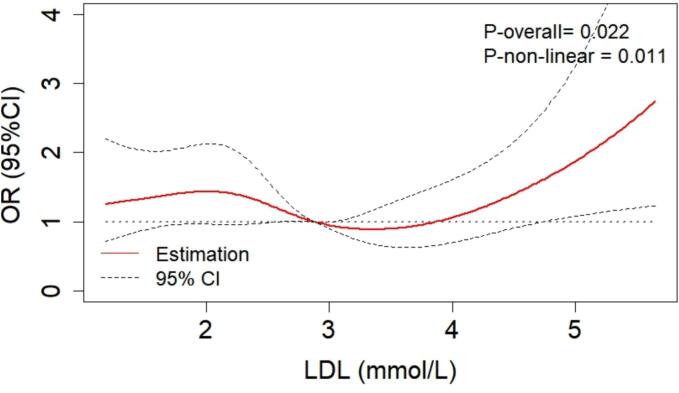
RCS analysis of non-linear association between LDL-C levels and diabetic kidney disease risk in Jinhua, China, combining data from 2017 and 2020–2021. This analysis was adjusted for age, gender, body mass index, hypertension, diabetic duration, angiotensin converting enzyme inhibitor/angiotensin receptor blocker usage, glycated hemoglobin, high-density lipoprotein cholesterol, and triglycerides. RCS: restricted cubic spline; LDL-C: low-density lipoprotein cholesterol; OR: odds ratio.

Generally, as shown in Fig. 3, the risk of DKD initially decreased, then gradually increased with higher LDL-C levels. After adjustment for age, gender, BMI, hypertension, diabetic duration, ACEI/ARB usage, HbA1c, HDL-C, and TG, a non-linear association between LDL-C levels and the risk of DKD (*P*_non-linear_ = 0.011) was observed based on RCS curve. The RCS curve’s shape was utilized to categorize LDL-C levels into three segments, elucidating the variability in DKD risk. Ultimately, the random forest algorithm identified two optimal cut-off points at 2.97 and 3.61 mmol/L.

### 3.5 Threshold effects analyses on association between LDL-C and DKD

In Table 3, the threshold effect analyses of LDL-C levels on DKD risk in T2D patients were presented, utilizing two optimal cutoff points at 2.97 and 3.61 mmol/L. After adjustment for the potential confounders, compared to patients with LDL-C levels in the range of 2.96–3.61 mmol/L, the DKD risk increased by 45 % in patients with LDL-C<2.96 mmol/L and by 47 % in patients with LDL-C>3.61 mmol/L. Relatively, patients with LDL-C>3.61 mmol/L had the highest DKD risk [1.47 (1.01–2.15), P=0.044]. Importantly, the association was notably stronger in females, showing ORs of 1.99 (1.21–3.27) for low LDL-C levels and 1.81 (1.01–3.25) for high LDL-C levels compared to moderate LDL-C levels.

**Table 3:** Threshold effect analyses of LDL-C on the risk of DKD among patients with T2D in Jinhua, China, combining data from 2017 and 2020–2021.

Variable	Univariate analyses		Multivariate analyses#	
	OR (95 % CI)	P value	OR (95 % CI)	P value
Overall				
LDL-C: 2.96–3.61 mmol/L	1.0 (reference)	−	1.0 (reference)	−
LDL-C: < 2.96 mmol/L	1.65 (1.25–2.16)	< 0.001	1.45 (1.08–1.96)	0.015
LDL-C:﹥3.61 mmol/L	1.48 (1.05–2.09)	0.026	1.47 (1.01–2.15)	0.044
P for trend	0.002		0.034	
Female				
LDL-C: 2.96–3.61 mmol/L	1.0 (reference)	−	1.0 (reference)	−
LDL-C: < 2.96 mmol/L	1.86 (1.21–2.87)	0.005	1.99 (1.21–3.27)	0.006
LDL-C:﹥3.61 mmol/L	2.06 (1.23–3.46)	0.006	1.81 (1.01–3.25)	0.046
P for trend	0.008		0.018	
Male				
LDL-C: 2.96–3.61 mmol/L	1.0 (reference)	−	1.0 (reference)	−
LDL-C: < 2.96 mmol/L	1.52 (1.07–2.16)	0.020	1.21 (0.82–1.78)	0.343
LDL-C:﹥3.61 mmol/L	1.11 (0.69–1.77)	0.680	1.22 (0.73–2.04)	0.448
P for trend	0.043		0.600	

# This analysis was adjusted for age, gender, body mass index, hypertension, diabetic duration, angiotensin converting enzyme inhibitor/angiotensin receptor blocker usage, glycated hemoglobin, high-density lipoprotein cholesterol, and triglycerides. T2D: type 2 diabetes; DKD: diabetes kidney disease; LDL-C: low-density lipoprotein cholesterol; OR: odds ratio.

### 3.6 Sensitivity analysis of non-linear relationships in DKD-classified patients

Sensitivity analysis was conducted using RCS analysis to validate the non-linear relationship between LDL-C levels and DKD risk among 854 patients who were clearly classified according to DKD criteria. A U-shaped non-linear relationship continued to exist, with a *P*_non-linear_ value of 0.013 (see Supplementary Fig. 1). The random forest algorithm identified two optimal cut-off points at 2.97 and 3.60 mmol/L.

## 4 Discussion

In this cross-sectional observational study of 1313 patients with T2D, we found that there was a non-linear association between the levels of LDL-C and the risk of DKD, the risk of DKD initially decreased, then gradually increased with higher LDL-C levels. Specifically, in comparison to patients with LDL-C levels between 2.96–3.61 mmol/L, DKD risk grew by 45 % in patients with LDL-C<2.96 mmol/L and by 47 % in those with LDL-C>3.61 mmol/L. Moreover, the relationship was noticeably more pronounced in female individuals.

Current therapeutic and/or preventive strategies for DKD primarily involved glycemic control, blood pressure management, inhibition of the renin-angiotensin system, and reduction of cardiovascular risks; nevertheless, these interventions did not effectively halt the advancement of DKD or the onset of end-stage renal disease (Liu et al., 2022). Hence, there was a crucial necessity to investigate another factors impacting DKD or potential targets for prevention and treatment beyond the existing therapeutic and preventive strategies. Dyslipidemia, a characteristic pathophysiological feature of DKD, disrupts lipid biosynthesis, transport, and clearance, resulting in increased levels of cholesterol, TG, low-density lipoproteins, and very-low-density lipoproteins in individuals with DKD (Elwakiel et al., 2024, Hager et al., 2017, Eid et al., 2019). This disruption leads to the abnormal accumulation of lipids in the kidneys, causing damage to renal cells and worsening the progression of DKD (Elwakiel et al., 2024, de Vries et al., 2014).

Evidence from both animal and human studies had suggested that dyslipidemia played a potential role in the development and progression of DKD (Zhou et al., 2023, Macisaac et al., 2014, Kelsey et al., 2022). Therefore, dyslipidemia, serving as both a risk factor and a potential target for treating DKD, prompted research on the clinical benefits of lipid-lowering medications (Zhou et al., 2023, Zhang et al., 2016). Zhou, et al. ((Zhou et al., 2023) evaluated 7272 individuals with statins usage and found that participants achieving intensive LDL-C control (<1.8 mmol/L) had a reduced risk of developing DKD compared to individuals with poor lipid control (LDL-C≥3.4 mmol/L) (HR 0.51, 95 % CI 0.32–0.81). However, a *meta*-analysis (Zhang et al., 2016) consisting of 23 clinical randomized controlled trials with 39,419 participants showed that the use of statins, primarily used for lowering TG and LDL-C, did not effectively slow the clinical progression of non-end stage CKD. The contradictory findings underscored the importance of further exploring the intricate and potentially complex relationship between dyslipidemia and DKD to improve the prognosis of patients with type 2 diabetes and globally reduce the burden of DKD on healthcare systems. Currently, TG levels had been consistently associated with an increased risk of DKD in previous studies (Li et al., 2023, Sacks et al., 2014, Yang et al., 2019). However, there was still controversy regarding the intrinsic association between LDL-C levels and DKD risk, necessitating further exploration. Therefore, this study conducted an investigation to determine the inherent association between LDL-C levels and DKD in patients with type 2 diabetes in China.

As demonstrated in Table 1 and Table 2 of this study, no statistical association was observed between LDL-C levels and DKD risk when LDL-C was considered as a continuous variable. This finding was consistent with several other studies (Lu et al., 2022, Chen et al., 2021, Ahn et al., 2020). However, conflicting results from some studies indicated that LDL-C levels in patients with DKD were significantly higher than those in patients without DKD (Leung et al., 2013, Coca et al., 2017). These discrepancies in research findings could be attributed to differences in study populations, variations in LDL-C measurement methods, or differential lipid metabolism profiles among the study populations. The findings from this study, as illustrated in Fig. 2 and Table 2, revealed statistical differences in eGFR levels, ACR, the prevalence, and the risk of DKD across different quartiles of LDL-C. These outcomes implied that the absence of a statistically significant correlation between LDL-C levels and DKD risk when treating LDL-C as a continuous variable might be linked to a non-linear association between LDL-C levels and DKD risk. Furthermore, This hypothesis received additional support from the RCS and the threshold effects analyses after adjustment for age, gender, BMI, hypertension, diabetic duration, ACEI/ARB usage, HbA1c, HDL-C, and TG. In this study, the confounders were selected based on previous research findings (Xu et al., 2021, Sacks et al., 2014) and clinical parameters in Table 1 that showed significant differences between the DKD and non-DKD groups. Levels of HDL-C and TG, which were closely associated with LDL-C, were also included in the model as confounding factors. However, the DKD assessment parameters, ACR and eGFR, were not considered as influencing factors for DKD. It is worth noting that the threshold effects of LDL-C levels on DKD risk were not confirmed in the male participants, but were more pronounced in the female subgroup. This might be due to the fact that the specific lipid profiles affected by steroid hormones differed between the sexes (Liang et al., 2023).

Misclassification bias is a significant factor that could impact the accuracy of the relationship under study. In the diagnosis of DKD, relying on a single time point measurement of ACR and eGFR may lead to incorrect classification of patient status, potentially misclassifying those without DKD as having the disease, or vice versa. This misclassification could result in biased estimates of the relationship between LDL-C levels and DKD risk. In particular, in this study, approximately 35 % of participants had only one measurement of ACR and eGFR, which increased the risk of misclassification. If the rate of misclassification of DKD status is high, it could attenuate or exaggerate the actual association between LDL-C levels and DKD risk. For example, misclassifying individuals at higher risk into lower risk groups could lead to an underestimation of the association between high LDL-C levels and increased DKD risk. To assess the impact of misclassification bias on the study outcomes, we conducted a sensitivity analysis on patients who were clearly classified according to DKD criteria, further validating the U-shaped non-linear relationship between LDL-C levels and DKD risk. The results showed a persistent U-shaped non-linear relationship between LDL-C levels and DKD risk, supporting our initial research findings. Additionally, the optimal LDL-C cut-off points identified through sensitivity analysis, at 2.97 mmol/L and 3.60 mmol/L, were almost identical with the results from the overall sample analysis, thereby enhancing the reliability of our study conclusions.

Several limitations should be acknowledged in the present study. First, kidney biopsy is considered the gold standard for diagnosing DKD. However, due to its invasive nature, many patients with diabetic renal impairment are either not eligible or are reluctant to undergo this procedure. This study relied solely on eGFR and ACR to assess DKD, and the data for about 35 % patients did not span three months. This approach might lead to underdiagnosis and misdiagnosis of DKD. Second, DKD is a complication in the late stages of diabetes, and it is a chronic process independent of the timing of diabetes diagnosis. Clinical diagnosis of DKD may not reflect early kidney damage, thus there may be misclassification of DKD in this study. Third, the research utilized a cross-sectional study design, indicating that causal relationships could not be inferred from the data alone. To establish causality between LDL-C levels and the risk of DKD, future longitudinal studies were essential. Fourth, the study population was derived from a single center in China, potentially constraining the generalizability of the findings to populations with diverse demographic and clinical profiles. Additionally, although efforts were undertaken to control for potential confounding factors, the existence of unmeasured confounders that could influence the outcomes could not be definitively excluded. Further studies included the more comprehensive analyses of confounding variables are needed to strengthen the reliability of the outcomes. Nevertheless, this study provided valuable insights into the relationship between LDL-C levels and the risk of DKD, establishing a basis for designing and conducting future prospective studies on this correlation.

## 5 Conclusion

We found that among the patients with T2D in China, the levels of LDL-C were non-linearly associated with the risk of DKD, patients with low (<2.96 mmol/L) or high levels (﹥3.61 mmol/L) of LDL-C had higher DKD risk than these with median levels. Additionally, the relationship was more pronounced in females. The significance of this study lies in revealing the varying risk of DKD associated with different levels of LDL-C in patients with T2D, highlighting the importance of identifying individuals at higher risk of developing DKD for timely intervention and management strategies based on their LDL-C levels.

## Funding

This research received the grant from Science Technology Department of Zhejiang province, China (LGF22H200021), Jinhua Science and Technology Bureau (2021-3-088, 2021-3-070), Zhejiang Medical and Health Science and Technology Project (2021KY384). The funders had no role in study design, data collection and analysis, decision to publish, or preparation of the manuscript.

## CRediT authorship contribution statement

**Xi Xiang:** Writing – original draft, Data curation, Conceptualization. **Guangming Chen:** Writing – original draft, Formal analysis. **Yongjun Ma:** Conceptualization. **Huabin Wang:** Writing – original draft, Formal analysis, Data curation, Conceptualization.

## Declaration of competing interest

The authors declare that they have no known competing financial interests or personal relationships that could have appeared to influence the work reported in this paper.

## Data Availability

Data are available on reasonable request from the corresponding author.
