# Supplementary material for: A non-linear association between low-density lipoprotein cholesterol and the risk of diabetic kidney disease in patients with type 2 diabetes in China
Source: Prev Med Rep. 2024 Jul 27;45:102840. doi: 10.1016/j.pmedr.2024.102840 (PMC11339054; doi:10.1016/j.pmedr.2024.102840)
Supplement: Supplementary Data 1 [file mmc1.doc]

**Supplementary figure 1**
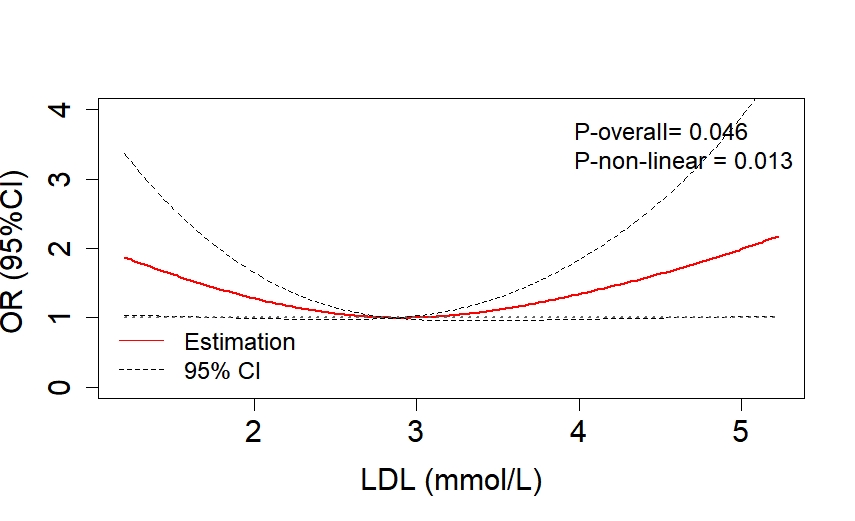
 **Sensitivity analysis of non-linear relationships among 854 clearly DKD-classified patients in Jinhua, China, combining data from 2017 and 2020-2021.** This analysis was adjusted for age, gender, body mass index, hypertension, diabetic duration, angiotensin converting enzyme inhibitor/angiotensin receptor blocker usage, glycated hemoglobin, high-density lipoprotein cholesterol, and triglycerides. DKD: diabetic kidney disease; RCS: restricted cubic spline; LDL-C: low-density lipoprotein cholesterol; OR: odds ratio.
